# AMPKβ isoform expression patterns in various adipocyte models and in relation to body mass index

**DOI:** 10.3389/fphys.2022.928964

**Published:** 2022-08-04

**Authors:** Franziska Kopietz, Eva Degerman, Olga Göransson

**Affiliations:** Department of Experimental Medical Sciences, Lund University, Lund, Sweden

**Keywords:** AMPKβ, adipocytes, obesity, expression, kinase activity, human

## Abstract

AMP-activated protein kinase (AMPK) activation is considered a useful strategy for the treatment of type 2 diabetes (T2D). It is unclear whether the expression and/or activity of AMPK in adipocytes is dysregulated in obesity. Also, the expression/activity pattern of AMPKβ isoforms, which are targets for AMPK activators, in adipocytes remains elusive. In this study we show that the two AMPKβ isoforms make roughly equal contributions to AMPK activity in primary human and mouse adipocytes, whereas in cultured 3T3-L1 adipocytes of mouse origin and in primary rat adipocytes, β1-associated activity clearly dominates. Additionally, we found that obesity is not associated with changes in AMPK subunit expression or kinase activity in adipocytes isolated from subcutaneous adipose tissue from individuals with various BMI.

## Introduction

AMP-activated protein kinase (AMPK) is a ubiquitously expressed protein, comprised of a catalytic α subunit and two regulatory subunits, β and γ. In mammalian cells, there are two isoforms of the α- and the β subunit, whereas the γ subunit exists in three different isoforms (Steinberg and Carling, 2019). Expression patterns of the specific isoforms can vary largely between cell types. The main role of AMPK is to sense and maintain cellular energy balance and its ability to do so is conferred by the capability of the γ-subunit to bind AMP, ADP, and ATP (Xiao et al., 2011; Chen et al., 2012; Herzig and Shaw, 2018). The binding of AMP/ADP induces phosphorylation of the catalytic α subunit at the activity-regulating site T172 by the upstream liver kinase B1 (Hardie and Carling, 1997; Woods et al., 2003; Carling, 2017). In addition to decreasing energy levels, AMPK is also activated by Calcium/Calmodulin Dependent Protein Kinase Kinase 2 in response to increasing levels of intracellular Ca^2+^ (Hawley et al., 2005; Hurley et al., 2005; Woods et al., 2005).

Previous studies showed that activation of AMPK could be beneficial in the treatment of type 2 diabetes (T2D) and insulin resistance (Merrill et al., 1997; Mu et al., 2001; Fryer et al., 2002; Foretz et al., 2005; Andreelli et al., 2006). A new generation of direct AMPK activators, like A-769662, 991, PF-06409577, and MK-8722, function in an AMP-independent way by binding to AMPK at the allosteric drug and metabolite (ADaM) site, located at the interface between the α- and β subunit (Cool et al., 2006; Goransson et al., 2007; Xiao et al., 2013; Cameron et al., 2016; Feng et al., 2018). The efficiency of these activators is highly dependent on the identity of the β isoform, with currently available compounds showing a varying preference for β1-containing AMPK-complexes (Xiao et al., 2013). Interestingly, long-chain fatty acids (LCFAs), known to be important regulators in cellular energy metabolism, have recently been reported as natural ligands of AMPK–also with a preference for AMPKβ1-containing complexes (Pinkosky et al., 2020). To be able to predict the effect of LCFAs and ADaM site binding compounds and thereby guide the future development of activators, it is warranted to carefully assess the contribution of the two β isoforms to AMPK activity in relevant tissues.

Adipocytes play an important role in the regulation of glucose- and lipid homeostasis, by for example maintaining insulin-sensitivity in liver and muscle (Smith and Kahn, 2016; Katwan et al., 2019). However, previous studies, by us and others, have come to inconclusive results about the distribution of the two AMPKβ isoforms in adipocytes (Kopietz et al., 2018; Katwan et al., 2019).

Obesity and the inability of adipose tissue to store excess energy is associated with an increased risk to develop systemic insulin resistance and T2D. Whether dysregulation of AMPK in adipose tissue plays a role in obesity-induced T2D in humans is only studied to a limited extent. AMPK activity in adipose tissue, measured as AMPK T172 phosphorylation, is decreased in morbidly obese, insulin resistant individuals compared to BMI-matched, insulin sensitive individuals (Gauthier et al., 2011; Xu et al., 2012). Furthermore, AMPK activity in subcutaneous adipose tissue is increased after gastric bypass surgery, also accompanied by increased phosphorylation of the AMPK downstream target acetyl-CoA carboxylase (ACC) at the inhibitory site S79 (Albers et al., 2015; Xu et al., 2015). It is important to stress that these previous studies on AMPK expression and activity in humans were performed on adipose tissue, containing several cell types other than adipocytes (Lee et al., 2013).

The aim of our study was to provide a more complete and quantitative assessment of the expression of the two AMPKβ isoforms and their contribution to total AMPK activity in various adipocyte models. Furthermore, we assessed AMPK activity as well as the expression of different AMPK subunit isoforms and downstream targets in human adipocytes in relation to BMI. Our results will contribute to a better understanding of AMPK expression in adipocytes from lean and obese individuals, facilitating future prediction of the response to AMPK ADaM site activators, and the possible role of AMPK dysregulation in obesity.

## Materials and methods

### Materials

Complete protease-inhibitor cocktail was from Roche (Mannheim, Germany). Pre-cast Novex SDS Polyacrylamide Bis-Tris gels, DTT and lithium dodecyl sulfate (LDS) sample buffer were purchased from Invitrogen (Carlsbad, United States ). Dulbecco’s Modified Eagle’s Medium (DMEM), phosphate-buffered saline (PBS), gentamicin and phenylisopropyl adenosine (PIA) were from Sigma Aldrich (St. Louis, MO, United States ). Protein-G-Sepharose was from GE Healthcare Biosciences (Uppsala, Sweden) and p81 phosphocellulose cation-exchange paper was from Whatman (Dassel, Germany). ^32^Pγ-ATP was obtained from Perkin Elmer (Boston, United States ). AMARA peptide (AMARAASAAALARRR) was synthesized by GL Biochem (Shanghai, China). Enhanced chemiluminescent (ECL) substrates SuperSignal West Pico and SuperSignal West Femto were obtained from Thermo Fisher Scientific (Rockford, IL, United States ).

### Antibodies

The following primary antibodies were used for Western blotting: anti-AMPK (#2603), anti-AMPK-pT172 (#2535; 1:1,000), anti-Raptor (#2280; 1:1,000), anti-Raptor-pS792 (#2083; 1:1,000), anti-ACC (#3662; 1:1,000), anti-ACC-pS79 (#3661; 1:1,000), anti AMPKβ1/β2 (#4150; 1:1,000), and anti-AMPKγ1 (#4187; 1:1,000) were all purchased from Cell Signaling Technology (Danvers, United States ) and anti-HSP90 from BD Biosciences (San Jose, CA, United States ). Antibodies used for immunoprecipitation: anti-AMPKα1, which were generated in house by immunizing rabbits with a peptide encompassing residues 344-358 of rat AMPKα1 (TSPPDSFLDDHHLTR) (Innovagen, Lund, Sweden); anti-AMPKβ1 (#27201), purchased from Signalway, and anti-AMPKβ2 (raised in sheep against residues 44-57 [SVFSLPDSKLPGDK] of rat β2), a kind gift from Professor D. Grahame Hardie (University of Dundee, United Kingdom). Anti-rabbit secondary antibodies conjugated to horseradish peroxidase (HRP) were from Thermo Fisher Scientific (Rockford, IL, United States ). Anti-mouse secondary conjugated to HRP was from GE Healthcare (Uppsala, Sweden).

### Isolation and lysis of primary rodent adipocytes

All animals were maintained in the animal facility at the Biomedical Centre, Lund University, Sweden and were housed in conventional shoebox cages with wood chip bedding, 2–4 (rats) or 4–6 (mice) animals per cage in a humidity-controlled room with 12 h light/dark cycle and non-restricted access to food and water. Adipocytes were isolated from epididymal or inguinal adipose tissue of 10- 12-weeks-old male C57BL/6J BomTac mice (Taconic Biosciences, Ejby, Denmark) or epididymal adipose tissue from 36- to 38-day-old Sprague-Dawley rats (Taconic Biosciences) via collagenase (1 mg/ml for epididymal, 1.5 mg/ml for inguinal tissue) digestion in a shaking incubator at 37°C. Digests were filtered and washed with Krebs-Ringer medium (120 mM NaCl, 4.7 mM KCl, 1.2 mM KH_2_PO_4_, 1.2 mM MgSO_4_) containing 25 mM HEPES pH 7.4, 200 nM adenosine, 2 mM glucose and 1% (w/v; rat) or 3% (mouse) BSA (KRH buffer). After isolation, the cells were washed in KRH buffer without BSA and lysed in 50 mM Tris-HCl pH 7.5, 1 mM EGTA, 1 mM EDTA, 1 mM sodium orthovanadate, 10 mM sodium-β-glycerophosphate, 50 mM NaF, 5 mM Na_4_P_2_O_7_, 0.27 M sucrose, 1 mM DTT, 1% (w/v) NP40 and complete protease inhibitor (1 tablet/50 ml) (lysis buffer). Lysates were centrifuged at 13,000 g for 15 min at 4°C and protein concentration in the supernatant was determined according to Bradford using BSA as standard (Bradford, 1976).

### Isolation and lysis of primary human adipocytes

**FIGURE 1:**
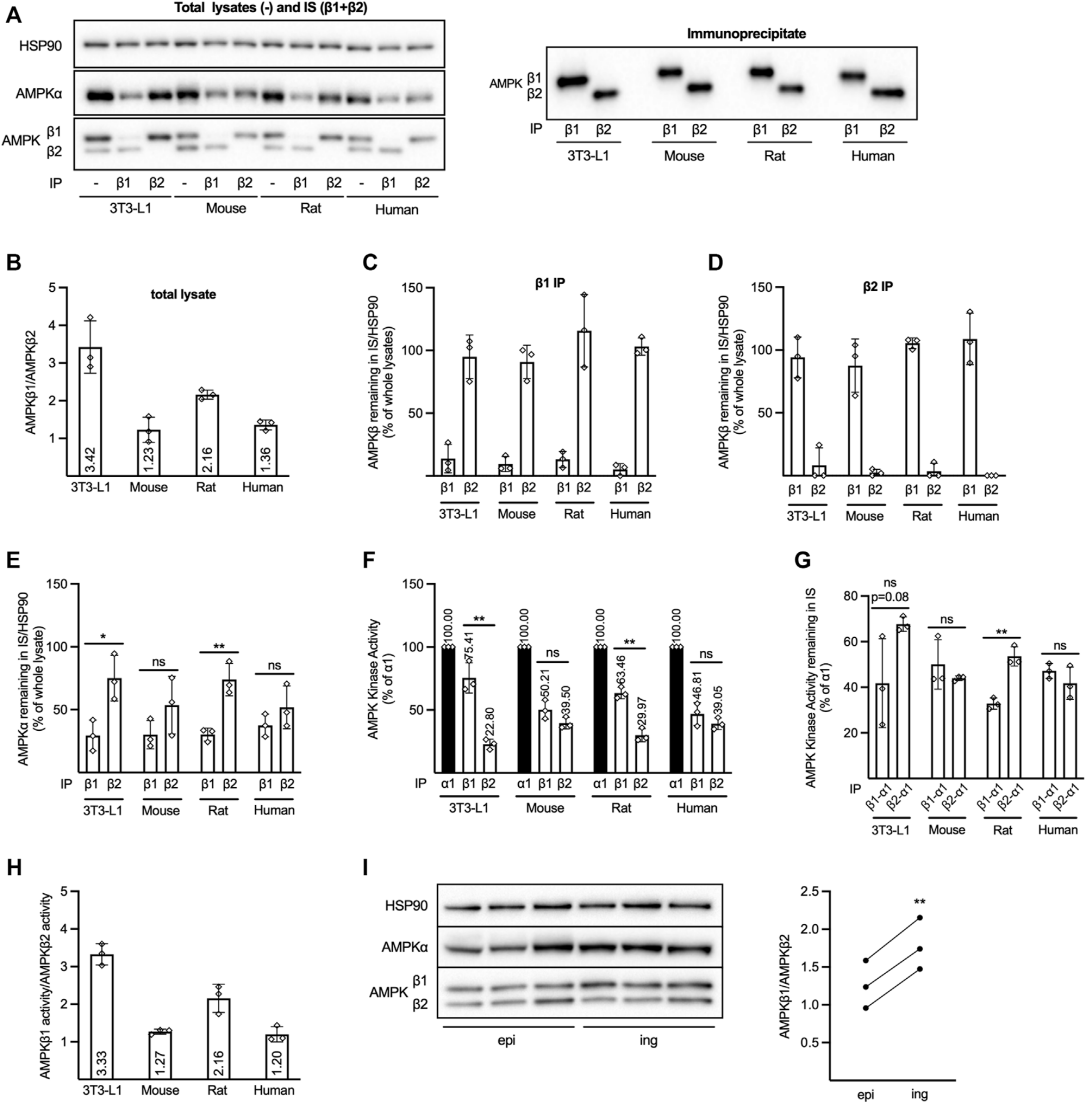
Expression of AMPKβ isoforms in different adipocyte models. Cell lysates were prepared from 3T3–L1 adipocytes or adipocytes isolated from mouse or rat epididymal- or human subcutaneous adipose tissue. **(A–E)** AMPKβ1 or β2 was immunoprecipitated (IP) from lysates using isoform-specific antibodies. Whole lysates ( = lysates prior to IP), IPs, and supernatants collected after IP (immuno-supernatants, IS), were analyzed by western Western blotting **(A)**. AMPKβ1 and β2 signals in whole lysates (B) or in immuno-supernatants (IS) collected after β1- **(C)** or β2 **(D)** IP were quantified and expressed as β1/β2 **(B)** or % of the corresponding signal from the whole lysate **(C, D)**. **(E)** Quantified AMPKα signals from immuno-supernatants (IS) collected after β1- or β2 IP were expressed as % of AMPKα in the whole lysate. **(F)** AMPK in vitro kinase activity was determined after α1- (black bar), β1- (left white bar) or β2 IP (right white bar). **(G)** AMPKα1 in vitro kinase activity was determined in immuno-supernatants (IS) collected after β1- or β2 IP in F. **(F, G)** Data is expressed as % of AMPKα1 activity in the lysate and absolute activity (mU/mg) is shown in Supplementary Figure S1. **(H)** Ratio of AMPKβ1 and β2-associated in vitro kinase activity determined in Supplementary Figure S1A. All data **(A–H)** is expressed as mean of three independent experiments ± SD. **(I)** AMPKβ1 and β2 in lysates of adipocytes isolated from mouse epididymal (epi) and inguinal (ing) adipose tissue from the same mouse (n = 3) were analyzed by western blotting (left panel) and quantified signals were expressed as β1/β2 (average epi = 1.26, average ing = 1.79; right panel). Statistical significance was determined by unpaired **(E–G)** or paired **(I)**, two-tailed, Students t-test * p 0.05, **pp 0.01, ns = non–significant.

**FIGURE 2:**
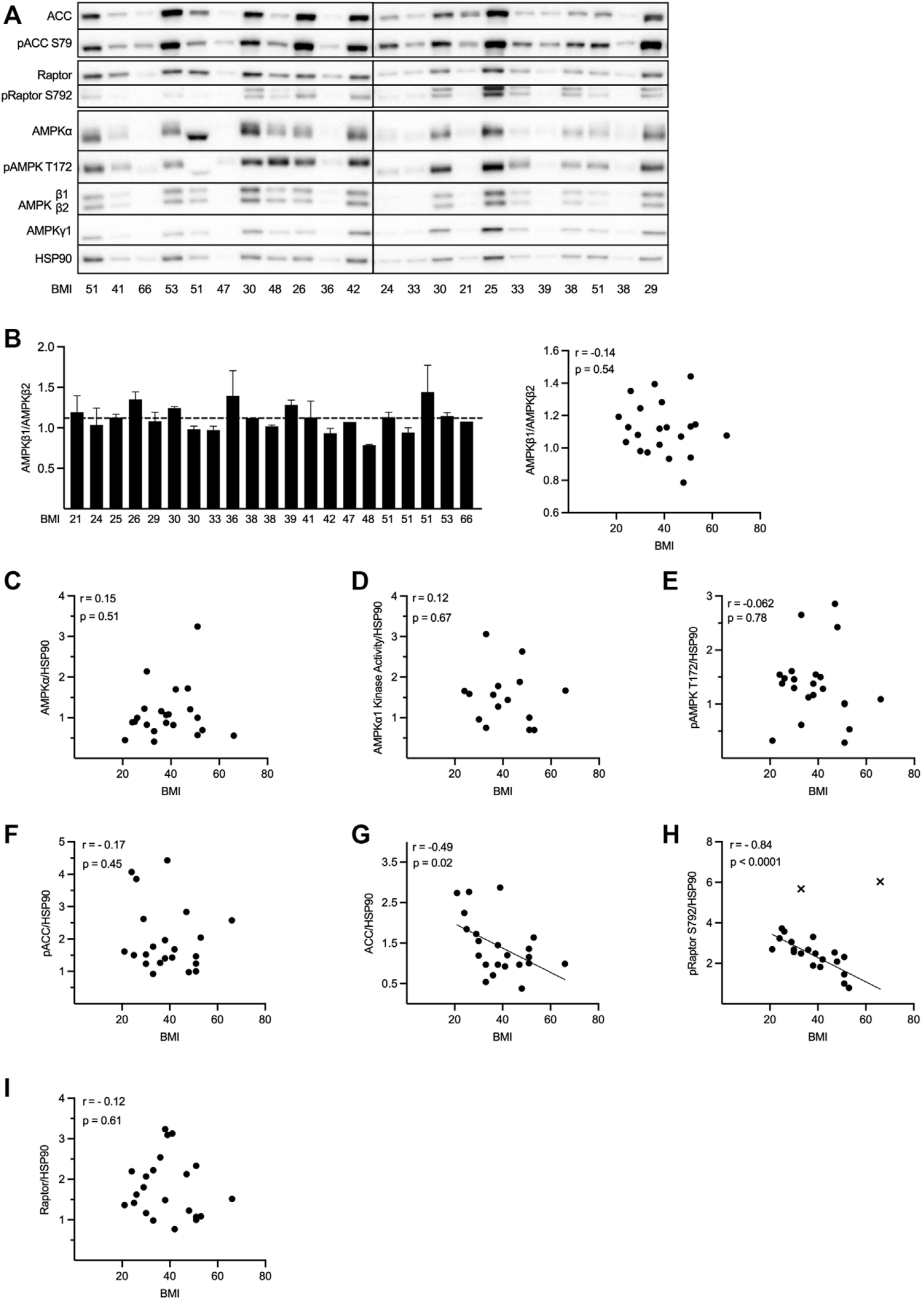
The expression and activity of AMPK is unchanged whereas Raptor S792 phosphorylation and expression of ACC are decreased in adipocytes from obese individuals. Primary adipocytes were isolated from human subcutaneous adipose tissue from 22 individuals (BMI 38.7 ± 11.5 kg/m^2^ [mean ± SD]; seven male, 15 female) and lysed immediately after isolation. AMPKα1 *in vitro* kinase activity measurements [for 15 (BMI 41.7 ± 11.5 kg/m^2^) out of the total 22 lysates] as well as Western blot analysis of AMPK subunit (α1, β1, β2, and γ1) expression and phosphorylation of AMPK T172 and AMPK downstream targets ACC at S79 and Raptor at S792 were performed. **(A)** Representative blots from two independent gel runs are shown. **(B)** AMPKβ1/β2 ratio for 21 out of 22 individuals (for one individual β1/β2 was undetectable in the WB; left panel) and plotted against BMI (right panel). Bar graph shows mean + SD of two individual gel runs. **(C–I)** AMPKα **(C)**, AMPKα1 *in vitro* kinase activity **(D)**, pAMPK T172 **(E)**, pACC S79 **(F)**, ACC **(G)**, pRaptor S792 **(H)**; excluded outliers are indicated as cross] and Raptor **(I)** levels normalized to HSP90 and plotted against BMI. Western blot signals normalized to HSP90, as well as kinase activity (1 corresponds to an activity of 44.5 mU/mg) were expressed as fold changes relative to one individual. Correlation analyses were performed using Pearson correlation test.

Human adipocytes used for the analysis of AMPKβ isoform expression (Figure 1) were isolated from abdominal subcutaneous tissue collected from female subjects who underwent reconstructive surgery [*n* = 3, BMI 23.4 ± 1.3 kg/m^2^ (mean ± SD)]. Cells were isolated and lysed as described for rodent adipocytes with the difference that after isolation, the cells were incubated overnight (o/n) in DMEM containing 0.1 mg/ml gentamicin, 3.5% (w/v) BSA and 200 nM PIA at 37°C under 5% CO_2_ before being washed in KRH buffer without BSA and lysed the next morning. Human adipocytes used for the analysis of AMPK expression and activity in relation to BMI (Figure 2) were isolated from abdominal subcutaneous tissue collected from 22 subjects who underwent laparoscopic cholecystectomy or gastric bypass surgery [BMI 38.7 ± 11.5 kg/m^2^ (mean ± SD); seven male, 15 female). After collagenase (1 mg/ml) digestion, the cells were washed in KRH buffer without BSA and homogenized in lysis buffer without NP-40 using a 0.4 mm needle. Homogenates were centrifuged at 100 g for 5 min at 4°C, the fat was removed and the infranatant solubilized by adding 1% (w/v) NP-40. After incubation on ice for 15 min, lysates were centrifuged at 13,000 g for 15 min at 4°C and the supernatant collected.

### Culture and lysis of mouse 3T3-L1 adipocytes

3T3-L1 fibroblasts were purchased from ATCC (Manassas, VA, United States ), and were cultured and differentiated to adipocytes as described previously (Berggreen et al., 2009). At day 8–12 post differentiation, cells of passages 10–15 were washed in PBS and lysed as described for primary cells.

### AMPKβ1 and -β2 immunoprecipitation (IP) for Western blotting

Lysates containing 50–100 μg of protein were incubated at 4°C for 1–2 h on a shaking platform with 25 µg AMPKβ1 or 5 µg AMPKβ2 antibody conjugated to 5 μl packed protein G-Sepharose beads. Subsequently, supernatants ( = immuno-supernatant; IS) were saved for further analysis and beads were washed twice with 500 μl lysis buffer supplemented with 0.5 M NaCl and 1 mM DTT, and twice with 500 μl of 50 mM Tris/HCl pH 7.5, 0.1 mM EGTA, and 1 mM DTT (Buffer A). LDS sample buffer (containing 75 mM DTT) was added to the beads, which were vortexed and centrifuged. Supernatants were transferred to new tubes ( = immunoprecipitate; IP). Remaining beads were discarded.

### Western blotting

Lysates, immuno-supernatants or IPs were heated in LDS sample buffer (75 mM DTT) and subjected to electrophoresis on pre-cast Novex 4%–12% Bis-Tris gels. Subsequently, proteins were electrotransferred to nitrocellulose membranes, which were then blocked for 30–60 min in 50 mM Tris-HCl pH 7.6, 137 mM NaCl and 0.1% (w/v) Tween 20 (TBS-T) containing 10% (w/v) skimmed milk and then probed with primary antibodies in TBS-T containing 5% (w/v) BSA for 16 h at 4°C. Protein detection was performed with HRP-conjugated secondary antibodies and ECL substrate. ECL signals were visualized using a ChemiDoc XRS + system followed by analysis of band intensities with the software Image Lab 6.1 (both from BioRad; Hercules, CA, United States ). For the analysis of AMPK expression and activity in relation to BMI, signals were normalized to an internal control loaded in the outer well of each gel and expressed as fold relative to one individual. Samples were run on gels twice, and were loaded in a different, randomized order, in each gel run.

### *In vitro* assay of AMPK activity

Lysates containing 5 μg (AMPKα1) or 20 µg (AMPKβ1 and β2) of protein were incubated at 4°C for 1–2 h on a shaking platform with 2 μg AMPKα1-, 25 µg AMPKβ1-or 5 µg AMPKβ2 antibody conjugated to 5 μl packed protein G-Sepharose. The IPs were washed twice with 500 μl lysis buffer supplemented with 0.5 M NaCl and 1 mM DTT, and twice with 500 μl buffer A. The kinase activity was measured in a volume of 50 μl containing 50 mM Tris-HCl pH 7.5, 0.1% 2-mercaptoethanol, 10 mM MgCl_2_, 0.1 mM EGTA, 0.1 mM ^32^Pγ-ATP (300 cpm/pmol) and 200 µM AMARA peptide for 20 min at 30°C. The assay was terminated by applying 40 μl of the reaction mixture on p81 cation-exchange phosphocellulose paper, followed by immersion of the paper in 50 mM phosphoric acid and 5-6 subsequent washes in 50 mM phosphoric acid before liquid scintillation counting. Incorporation of ^32^P-phosphate was expressed as pmol ATP incorporated/mg protein/min (mU/mg).

### Statistical analysis

Statistical and correlation analyses were made with GraphPad 9 (La Jolla, CA, United States ) using unpaired or paired, two-tailed, Student’s *t*-test (Figure 1) and Pearson correlation test (Figure 2, Supplementary Figure 2), respectively. Statistical significance levels: *p < 0.05, **p < 0.01, ns = non-significant. Correlation levels: r = 0, none; r < ± 0.29, weak/negligible; r ± 0.30—± 0.49, moderate; r ± 0.5—± 0.99 strong; ± 1, perfect.

## Results

### Expression of AMPKβ isoforms in different adipocyte models

To determine the respective contribution of β1-and β2-containing AMPK complexes to total AMPK activity in various commonly used adipocyte models, Western blot analysis and *in vitro* kinase activity measurements were performed. Initially, total cell lysates (Figure 1A, left panel), AMPKβ1-or β2 immunoprecipitates (IPs) (Figure 1A, right panel), as well as supernatants collected after IP (immuno-supernatants; IS) (Figure 1A, left panel), were analyzed by Western blotting. When using an AMPKβ1/β2 pan antibody, targeting the sequence surrounding His233, which is conserved between the isoforms as well as species, for Western blotting of total cell lysates our results are in line with what we previously reported (Kopietz et al., 2018). As shown in Figure 1A, primary mouse and human adipocytes display a more even level of the two β isoforms compared to cultured mouse 3T3-L1 (mouse origin) or primary rat adipocytes, in which the β1 signal is 2–3 times higher than for β2 (Figure 1A, left panel, B). IP with an AMPKβ1-specific antibody removed almost all β1 from human lysate (5.2% remaining), whereas a small amount remained in 3T3-L1 (13.8%), mouse (9.4%) and rat adipocyte lysates (13.1%) (Figure 1A, left panel, C). After IP with an AMPKβ2-specific antibody, 8.1% of the AMPKβ2 was detected in the 3T3-L1 immuno-supernatant, whereas the IP was virtually 100% efficient in the other cell types (Figure 1A, left panel + D). No β2 was detected in the β1 IP and vice versa (Figure 1A right panel), demonstrating the isoform specificity of the antibodies. Collectively, this shows that our IP protocols were efficient and specific. Quantification of the catalytic AMPKα subunit revealed that less AMPKα remained in the immuno-supernatant after β1 IP than after β2 IP in 3T3-L1 and rat adipocytes, whereas there was no statistically significant difference in the amount of AMPKα remaining after β1-and β2 IP from mouse or human adipocytes (Figure 1A left panel, E). We next analyzed *in vitro* AMPK kinase activity in β1 and β2 IPs (Figure 1F), as well as AMPKα1 activity in the immuno-supernatants after β1 (“β1-α1”) and β2 (“β2-α1”) IP (Figure 1G). All activities were compared to total AMPKα1 kinase activity in the lysates (black bars, Figure 1F), as α1 has earlier been shown to be the predominant α isoform responsible for AMPK activity in adipocytes (Lihn et al., 2004; Daval et al., 2005; Kopietz et al., 2018; Katwan et al., 2019). Indeed, the combined β1 and β2-associated AMPK activity did not exceed AMPKα1 activity in any of the models (Figure 1F), indicating that there is little AMPKα2 activity in these cells. Similar to western blot data using the pan AMPKβ1/β2 antibody (Figures 1A,B), and in line with the amount of AMPKα1 which was shown to be bound to the respective β isoform (Figure 1E), our *in vitro* kinase activity analysis indicated that primary mouse and human adipocytes contained roughly equal amounts of β1 and β2-associated AMPK activity, whereas in 3T3-L1 and rat adipocytes, β1-was markedly higher than β2 activity [Figure 1F (activity expressed as % of AMPKα1 in the lysate), Supplementary Figure S1A (activity expressed as mU/mg)]. Similar to the western blot data in Figure 1E, less AMPKα1 activity remained in the immuno-supernatant after β1 IP than after β2 IP in rat adipocytes, whereas the amount of AMPKα1 activity remaining after β1-or β2 IP from mouse or human adipocytes was roughly the same (Figure 1G). The activity remaining after β1 IP in 3T3-L1 adipocytes was lower in each experiment but varied and was not significantly different to that after β2 IP (Figure 1G). Additionally, the ratio of AMPKβ1-to AMPKβ2-associated *in vitro* kinase activity (calculated using activities presented in Figure 1F and Supplementary Figure S1A) (Figure 1H) was virtually identical to the one we obtain using the AMPKβ1/β2 pan antibody for Western blotting (Figure 1B). In order to also address a possible variation in the expression of the AMPKβ isoforms between different adipose tissue depots, we have performed a comparison between mouse epididymal and inguinal fat–the latter representing a subcutaneous depot. As shown in Figure 1I, the AMPKβ1/β2 ratio was slightly, but consistently higher in the inguinal depot.

### The expression and activity of AMPK are unchanged, whereas Raptor S792 phosphorylation and expression of ACC are decreased in adipocytes from obese individuals

*In vitro* kinase activity of AMPKα1 as well as protein and phosphorylation levels of AMPK subunits and its substrates ACC and Raptor were assessed in primary human subcutaneous adipocytes isolated from 22 individuals with a BMI in the range of 21–66 kg/m^2^ (Figure 2). A strong positive correlation between pT172 and AMPKα1 *in vitro* activity, ACC S79 and Raptor S792 phosphorylation in each individual was observed, regardless of BMI (Supplementary Figures S2A–C). Furthermore, the analysis revealed a strong positive correlation of AMPK T172 phosphorylation to the expression of the catalytic AMPKα subunit (Supplementary Figure S2D), as well as between AMPKα and the regulatory β1-, β2-and γ1 subunits (Supplementary Figures S2E–G). These expected correlations provide a validation of the experimental approach in this part of the study.

Next, we assessed whether obesity/BMI is associated with altered AMPK expression and/or activity. To be able to compare the different individuals, quantified Western blot signals and kinase activities were normalized against HSP90. Expression of HSP90 varied considerably, however, these differences were not related to the BMI (Supplementary Figure S2H). In line with the data presented in Figure 1, suggesting roughly equal expression of the two AMPKβ isoforms in human adipocytes, analysis of AMPKβ1 and -β2 in 21 individuals (AMPKβ levels in one individual were too weak for analysis) with varying BMI revealed an average AMPKβ1/β2 ratio of 1.1 (Figure 2B, left panel; average indicated by dotted line). No sex-dependent differences in the AMPKβ1/β2 ratio were detected (Supplementary Figure S2I) and neither the variation in the AMPKβ1/β2 ratio (Figure 2B, right panel), nor the AMPKβ1 or -β2 levels themselves (Supplementary Figures S2J, K), were found to be correlated to the BMI. A great variation of AMPKα levels (Figure 2A, C), as well as AMPKα1 *in vitro* kinase activity (Figure 2D) was observed when comparing individuals with various BMI. However, neither AMPKα expression (Figure 2C) nor kinase activity (Figure 2D) or AMPK T172 phosphorylation levels (Figure 2E) correlated with BMI. In line with these results, ACC S79 phosphorylation did not change with increasing BMI (Figure 2F). Interestingly however, we observed a moderate negative correlation between BMI and ACC levels (Figure 2G), indicating a decrease in ACC with increasing BMI. Somehow unexpectedly, when analyzing another AMPK substrate, a strong negative correlation between Raptor S792 phosphorylation and BMI (after outlier exclusion; Figure 2H) was observed. In contrast to ACC, there was no correlation between BMI and Raptor expression (Figure 2I).

## Discussion

Although it has to some extent been studied previously by us and others, the contribution of β1 and β2 isoforms to AMPK activity in adipocytes remains unclear (Kopietz et al., 2018; Katwan et al., 2019). In the current study, we used a quantitative approach to determine the absolute activity of AMPKβ in different adipocyte models and showed that in primary mouse and human adipocytes, the levels of AMPKβ1 and β2-containing complexes are roughly equal, whereas in 3T3-L1 adipocytes and primary rat adipocytes, the β1-containing complexes are predominant. Mouse adipocytes might thus be a better model than rat adipocytes when wishing to translate results regarding AMPK to humans. Another important finding is that the β1/β2 ratio obtained using Western blotting with the AMPK pan β antibody closely reflected the one generated with IP kinase assay. The implication of this is that the pan antibody can be used in the field to reliably determine β1/β2 ratios in other tissues/models.

In our study from 2018, the β isoform distribution in different adipocyte models was only investigated by western blotting of whole cell lysates using the AMPKβ1/β2 pan antibody (Kopietz et al., 2018). When using isoform pan antibodies, one needs to consider the possibility that the antibody binds the two isoforms with different efficiencies, and it was therefore only possible to compare the relative amounts of β1 and β2 between the different adipocyte models, and not the actual levels, or β1 in relation to β2 in each model. To be able to analyze the contribution that each isoform makes in each of the adipocyte models, we now employed IP. Using Western blotting, we first confirmed that our immunoprecipitating β antibodies were isoform-specific and that they had the same high level of IP efficiency. In the same experiments, we assessed the amount of AMPKα bound to the respective β isoform. Moreover, we employed IP followed by *in vitro* kinase activity to compare the actual levels of β1-and β2-associated activity, and how much they contribute to total AMPKα1 activity. In these ways, we identified AMPKβ1-containing complexes as the most prevalent in 3T3-L1 and rat adipocytes, while in mouse and human adipocytes, β1-and β2-containing complexes made roughly equal contributions, with only slightly more β1 than β2. The conclusion from our IP analysis is strengthened by the fact that the results are line with those obtained using an independent method, namely Western blotting with the pan AMPKβ antibody, which was used both in our current and previous study. Indeed, β1/β2 ratios generated with the pan antibody were virtually identical to those obtained in the IP analysis. This indicates that the pan antibody has similar affinity for the two isoforms and can indeed be used to assess relative isoform expression in various models.

Our results contradict those published by Katwan et al. (2019) who concluded that AMPKβ2 is the predominant isoform in 3T3-L1-, isolated rat- and mouse adipocytes and in human subcutaneous adipose tissue. The reason for this discrepancy is not clear but could be due to different specificity and/or IP efficiency of the antibodies used. In our study, we directly tested the efficiency of the AMPKβ antibodies using western blotting of the immunodepleted supernatants and found the IP to be almost quantitative, and if anything, slightly more efficient for β2. This analysis was performed in the same adipocyte models which were also analyzed for AMPK activity, and moreover confirmed that the antibodies were entirely isoform specific.

Taken together, our results indicate that mouse adipocytes are the most similar to human adipocytes, regarding AMPKβ1/β2. However, one needs to be aware of potential differences between fat depots, since we used epididymal for mouse/rat and subcutaneous for human, in the species comparison. Our comparison of mouse depots showed a higher AMPKβ1/β2 ratio in inguinal than in the epididymal fat, the former being a subcutaneous—and the latter more of a visceral depot. Based on this result one could speculate that the AMPKβ1/β2 ratio might be even lower in human visceral than in subcutaneous fat. It is however important to note that the epididymal depot is not a pure visceral depot and one needs to be careful when making such assumptions. The roughly equal contribution of the two β isoforms in human adipocytes might also imply an importance of utilizing pan β isoform AMPK activators if the aim is to achieve maximal AMPK activation in these cells. Moreover, considering the differential contribution of the two β isoforms to total AMPK activity in the different cell models, one might expect differential sensitivity to β1-selective vs. pan AMPK activators in these models. However, no systematic comparison has been reported so far. A critical focus for future studies should be possible (β) isoform-specific roles of AMPK, since this would profoundly affect the outcome of AMPK activation using isoform-selective compounds. It would for example be interesting to investigate whether AMPKβ1-and β2-containing complexes have different functions in the regulation of adipocyte metabolism and thus possible consequences/roles of the β1 predominance in 3T3-L1 and rat adipocytes.

Variations in the relative AMPKβ1/β2 expression could in turn result in inter-individual differences in the response to isoform-selective AMPK activators, in particular if AMPKβ1/β2-containing complexes have different functions. Therefore, we investigated this possibility, as well as the expression and regulation of the AMPK pathway as a whole, in human adipocytes in relation to BMI. Previous studies investigating AMPK activity and regulation in human adipose tissue were focused on morbidly obese individuals alone, comparing insulin sensitive versus insulin resistant individuals, as well as changes induced by gastric bypass surgery (Gauthier et al., 2011; Xu et al., 2012; Albers et al., 2015; Xu et al., 2015). However, the association between BMI and AMPK activity/expression remained unclear so far. Furthermore, all previous studies investigated adipose tissue, containing various other cell types than adipocytes, suggesting that the signals obtained did not necessarily originate from adipocytes.

In line with the data obtained when comparing different adipocyte models, our results from 21 human individuals with varying BMI showed a fairly stable β1/β2 ratio of around 1—as determined using the pan β antibody. This data indicates that the ability of isoform selective compounds to activate AMPK in adipocytes will most likely be similar, regardless of BMI of the subject. We also did not find any significant differences in the β1/β2 ratio between men and women. However, we interpret this result with caution since these two groups were small (number of males = 6) and not matched, for example with regard to age and BMI.

Furthermore, analysis of AMPKα expression, AMPKα1 activity and T172 phosphorylation showed a great variation among individuals, without any correlation to BMI. Previous studies showed increased AMPK T172 phosphorylation in adipose tissue after weight loss as a result of gastric bypass surgery—an observation which was also accompanied by decreased malonyl-CoA levels–indicating decreased ACC activity (Albers et al., 2015; Xu et al., 2015). Taken together, these previous observations pointed towards a negative association between weight (BMI) and AMPK activity in adipose tissue. As adipose tissue also contains several other cell types, such as fibroblasts, endothelial- and immune cells (Lee et al., 2013), one interpretation of our results is that the previously observed changes in AMPK activity were attributable to AMPK in non-adipose cells. Alternatively, the changes in AMPK activity observed in previous studies could be linked to the loss of weight, rather than the weight *per se*.

In line with our observation of a negative correlation between BMI and ACC levels in isolated adipocytes, Albers et al. (2015) reported increased ACC levels post bariatric surgery, suggesting an elevation of ACC levels with weight loss. These observations suggest that ACC levels indeed decline with increasing BMI, which could possibly contribute to the reduced *de novo* lipogenesis which is observed in insulin resistance and obesity (Smith and Kahn, 2016). Interestingly, we also observed a negative correlation between the phosphorylation of the AMPK downstream target Raptor and BMI, although no BMI-dependent changes in AMPK activity were detected. However, considering shown compartmentalization of AMPK, one can speculate that the activity of a distinct pool of AMPK, which is regulating Raptor phosphorylation, might indeed be altered in a BMI-dependent way (Miyamoto et al., 2015; Zong et al., 2019). Moreover, it is possible that allosteric regulation, for which we cannot account in the *in vitro* setting, but that occurs *in vivo*, results in the observed changes in Raptor phosphorylation in relation to BMI. Raptor is required for assembly and substrate binding of the mammalian target of rapamycin complex 1 (mTORC1), which is known to stimulate cell growth/proliferation and inhibit autophagy when active (Laplante and Sabatini, 2012). Furthermore, it has been shown that high levels of insulin and nutrients, as found in the obese state, promote mTORC1 activity which in turn is suggested to cause ribosomal protein S6 kinase beta-1 (S6K1)-mediated inhibition of insulin signaling (Laplante and Sabatini, 2012). As AMPK-dependent phosphorylation of Raptor has earlier been shown to be required for the inhibition of mTORC1 in the event of energy stress, decreased phosphorylation with increasing BMI is in line with the reported increase of mTORC1 activity in obesity (Gwinn et al., 2008; Laplante and Sabatini, 2012). However, as AMPK activity remained unaltered with increasing BMI, the underlying cause of the decreased Raptor S792 phosphorylation remains elusive.

Considering the previous studies showing decreased AMPK activity in insulin resistant, compared to BMI-matched insulin sensitive individuals (Gauthier et al., 2011; Xu et al., 2012), it would be interesting to study AMPK signaling in adipocytes also in relation to insulin sensitivity. Moreover, as the material used here only included few non-obese subjects (5 out of 22), it would be valuable to compare the observations made here, to non-obese individuals with varying BMI.

In summary, we have shown that the expression and contribution to total cellular AMPK activity of the two regulatory AMPKβ isoforms varies among different adipocyte models, and that β1 and β2 isoforms show a roughly equal contribution in human adipocytes. Furthermore, we showed that human obesity is not associated with changes in AMPK expression or activity in adipocytes but revealed a negative correlation between BMI, the expression of ACC and phosphorylation of Raptor on S792.

## Data Availability

The raw data supporting the conclusion of this article will be made available by the authors, without undue reservation.
