# Supplementary material for: AMPKβ isoform expression patterns in various adipocyte models and in relation to body mass index
Source: Front Physiol. 2022 Aug 4;13:928964. doi: 10.3389/fphys.2022.928964 (PMC9386264; doi:10.3389/fphys.2022.928964)
Supplement: Supplementary file 1 [file DataSheet1.pdf]

Supplementary Material

Kopietz et al. 2022

Supplementary Figure 1

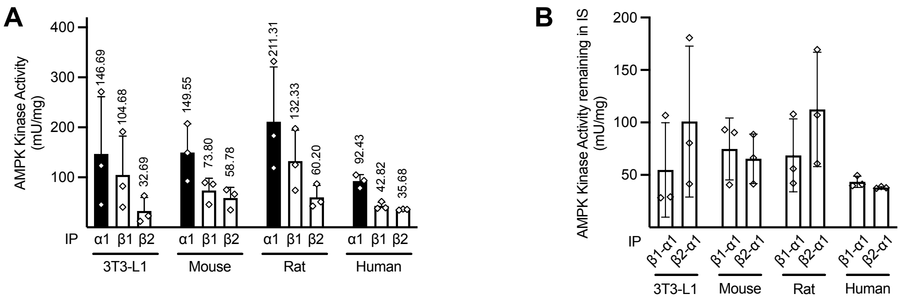

Supplementary Figure 1. AMPK in vitro kinase activity shown in Figure 1F+G presented as absolute values (mU/mg). Bar graph shows the mean of three independent experiments +SD with **(A)** corresponding to Fig. 1F and **(B)** to Fig. 1G.

Supplementary Figure 2

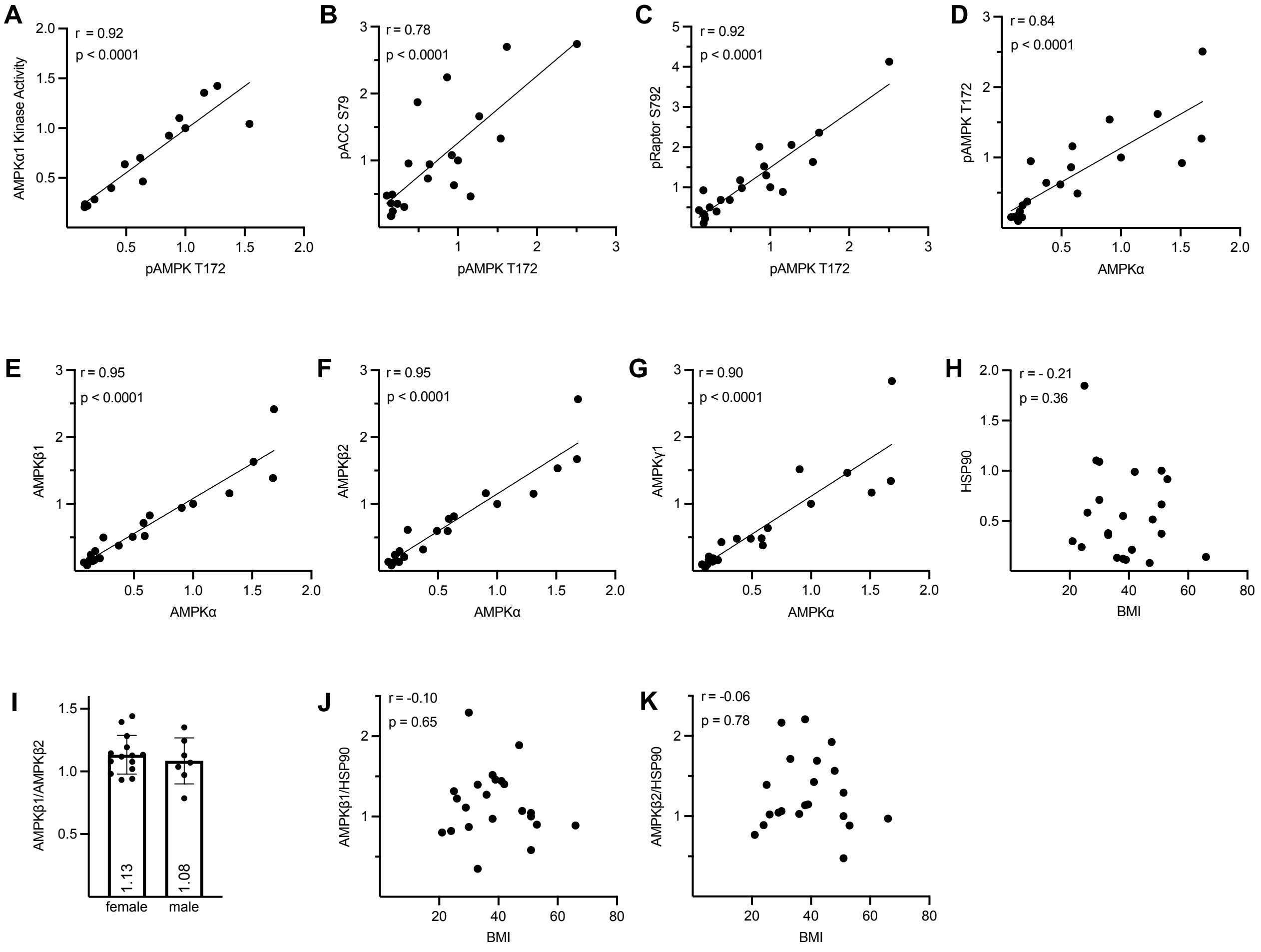

Supplementary Figure 2. Complementary analysis of AMPK activity and isoform expression in adipocytes isolated from humans with various BMI. Data shown is based on the same analyses as shown in Fig 2. **(A-C)** AMPK T172 phosphorylation levels were plotted against AMPKα1 *in vitro* kinase activity **(A)**, pACC S79 **(B)** and pRaptor S792 **(C)**. **(D-G)** AMPKα signals plotted against pAMPK T172 **(D)**, AMPKβ1 **(E)**, AMPKβ2 **(F)** and AMPKγ1 **(G)**. **(H)** HSP90 levels plotted against BMI. **(I)** AMPKβ1/β2 ratio grouped according to sex. **(J-K)** HSP90-normalized AMPKβ1 **(J)** and AMPKβ2 **(K)** levels plotted against BMI. Kinase activity (**A**, for 15 [BMI 41.7±11.5 kg/m^2] out of the total 22 lysates; 1 corresponds to an activity of 44.5 mU/mg) as well as western blot signals **(A-H, J+K)** were expressed as fold changes relative to one individual. Correlation analyses were performed using Pearson correlation test.
